# Disclosing Child Sexual Abuse to a Health Professional: A Metasynthesis

**DOI:** 10.3389/fpsyt.2022.788123

**Published:** 2022-06-03

**Authors:** Emilie Manolios, Ilan Braoudé, Elise Jean, Thomas Huppert, Laurence Verneuil, Anne Revah-Levy, Jordan Sibeoni

**Affiliations:** ^1^ECSTRA Team, UMR-1153, Inserm, Universiteì de Paris, Paris, France; ^2^AP-HP, Service de Psychiatrie et Addictologie de l’Adulte et du Sujet Âgé, Hôpital Européen Georges-Pompidou, Paris, France; ^3^Service Universitaire de Psychiatrie de l’Adolescent, Argenteuil Hospital Centre, Argenteuil, France; ^4^Pôle Précarité, GHU Paris Psychiatrie and Neurosciences, Paris, France

**Keywords:** child sexual abuse, disclosure, qualitative research, systematic review, metasynthesis

## Abstract

**Objective:**

Sexual abuse is a major public health problem. Its disclosure to a health professional could help to reduce its impact on survivors’ lives. The objective of this metasynthesis, combining a systematic review and an analysis of the qualitative studies, was to explore the qualitative literature concerning the experience of a survivor disclosing sexual violence experienced in childhood to a health professional, from the perspective of both.

**Methods and Data Sources:**

We used four databases and two journals (Medline, PsycINFO, EMBASE, and SSCI, and the Journal of Sexual Abuse and Child Abuse and Neglect) to identify studies concerning this disclosure of sexual abuse to healthcare professionals from the point of view of the survivors and the health professionals. After assessing the methodological quality of the articles with the “Critical Appraisal Skills Program (CASP),” we conducted a thematic analysis of the data extracted during the review.

**Results:**

This review includes 20 articles, covering the data of 612 participants: 291 who were adults at the time of the study but abused in childhood, 152 minors, 14 parents of adolescents, and 155 healthcare professionals. Two themes emerged from the analysis: (1) the disclosure as experienced by the professionals, and (2) the disclosure as experienced by the survivors.

**Conclusion:**

Our results show that survivors had a diachronic approach to the experience of disclosure. They suggest a change over time in how survivors experience disclosure: relief and release were seen only among the adult participants, at a distance from — long after — the disclosure. This study made it possible to identify new perspectives for research in the field of child psychiatry and to formulate concrete clinical proposals, in particular, by applying the principle of patient experts to involve now-adult survivors in training and increase the awareness of the healthcare professionals concerned.

## Introduction

Early detection by healthcare professionals of child sexual abuse (CSA) is a major public health issue (WHO) (1). A 1994 literature review of 39 studies from 21 countries reported a prevalence below 10% in boys and between 10 and 20% in girls (2). A 2011 study estimates that 18% of girls and 8% of boys worldwide have experienced sexual abuse (1). The prevalence among patients receiving care by psychiatrists and child psychiatrists is likely to be still higher (3).

Numerous studies have documented the impact of sexual abuse at psychiatric (4–6), somatic, social (7), and even subclinical (8) levels. The disclosure of such abuse is increasingly better documented: the process of disclosure, the choice of person whom the survivor tells (family, friends, school, or health professionals), the consequences of disclosure (9, 10)—both positive, especially in terms of prevention [preventing a phenomenon of revictimization (11) or neurological deterioration (8)], and negative [often related to the environmental response (12)], as well as the time interval between the abuse and the disclosure. This delay on average can extend for 20–46 years (13–15). Moreover, the expression “early disclosure” is used for disclosure that occurs within 15 years after the abuse (16). Furthermore, it is estimated that 20% of women and 39% of men survivors never tell anyone. Only 16–25% of survivors sexually abused in childhood or adolescence disclose it before adulthood (17).

Many qualitative studies have examined the disclosure of CSA. They explore the experience of survivors, their families, and the professionals involved. Qualitative methods are particularly pertinent in this context. Moreover, the WHO recently affirmed the importance of synthesizing data from qualitative studies to help develop health policy and clinical practices (18). Metasynthesis is thus a method of choice for producing new knowledge from the analysis of qualitative literature (19, 20).

To our knowledge, two reviews of the literature on the topic of disclosure of sexual abuse to professionals have already been published. Alaggia et al. (21) identified 11 quantitative or mixed studies and 22 qualitative studies. They uncovered factors that promoted disclosure, such as the desire to protect other potential victims, or factors specific to healthcare professionals (a supportive investigator who listens, believes the discloser, and creates a safe environment), and those that impede disclosure, such as being a boy, the doctor’s position of authority, or an emergency situation. Watkins-Kagebein et al. (22) conducted a metasynthesis of seven qualitative studies, based on accounts of sexual violence by child and adolescent survivors. They described different forms of disclosure—integrated in the care process and distressing to the survivors—and concluded that supplementary qualitative research is needed to explore its intersubjective, social, and cultural aspects.

To our knowledge (after performing this systematic review), no literature review or metasynthesis specifically focuses on disclosure to a health professional. The objective of this study was to conduct a metasynthesis of the qualitative studies exploring the experience of disclosing CSA to a health professional, from the point of view of the survivors, but also of the professionals, to cross these different points of view to generate new knowledge and uncover new perspectives for clinical practice and research.

## Materials and Methods

### Study Design

This metasynthesis is based on the meta-ethnographic model and follows the thematic synthetic procedures described by Thomas and Harden (20). It meets the ENTREQ requirements (23). Our procedure took place in six successive stages:

Definition of the research question, participants, and types of studies to include
Identification and selection of studies
Evaluation of the studies’ quality
Analysis of the studies, identification of their themes and translation of these themes through each study
Generation of the analytic themes and configuration of the synthesis
Production of a written synthesis.

The thematic analysis covers two phases: a descriptive phase during which we defined and compared themes and an interpretative phase for developing original results.

### Research Strategy

Four databases as well as two journals were systematically queried: MEDLINE, PsycInfo, CINAHL, SSCI, and the *Journal of Sexual Abuse* and *Child Abuse and Neglect* from February through June, 2019, with an update in January 2021. Preliminary research had identified several articles to enable the selection of key words and the construction of algorithmic search phrases. Table 1 details this research strategy.

**TABLE 1:** Algorithm for complete search of each database.

Database	Phrase
Pubmed	(((((“report*” OR “Mandatory Reporting”(Mesh) OR “Self Disclosure”(Mesh) OR “Disclosure”(Mesh) OR “Truth Disclosure”(Mesh) OR “Attitude of Health Personnel”(Mesh) OR “Interview, Psychological”(Mesh) OR “Interviews as Topic”(Mesh) OR “Health Knowledge, Attitudes, Practice”(Mesh) OR “disclos*” OR “identif*” OR “screen*” OR “assess*” OR “elicit*”)) AND (“Child” (Mesh) OR “Adolescent” (Mesh) OR “adolesc*” OR “young” OR “youth” OR “infant” OR “school age” OR “preschool” OR “child*” OR “teen*”)) AND (“Child Abuse, Sexual” (Mesh) OR “sexual violence” OR “Child abuse” OR “sexual abuse” OR “sexual exploitation” OR “sexual assault” OR “molestation” OR “incest”)) AND (“perception” OR “attitude” OR “feeling” OR “knowledge” OR “lived experience” OR “belief” OR “view” OR “perspective” OR “opinion” OR “experience” OR “image” OR “Attitude to Health” (Mesh) OR “Knowledge” (Mesh) OR “Psychology” (Mesh) OR “Self Concept” (Mesh) OR “Health Services Administration” (Mesh))) AND (“qualitative research” (Mesh) OR “Nursing Methodology Research” (Mesh) OR “Focus Groups” (Mesh) OR “observation” (Mesh) OR “qualitative research” OR “qualitative study” OR “qualitative method” OR “grounded theory” OR “interview” OR “qualitative approach” OR “qualitative analysis”)
PsycINFO	(DE “Qualitative Research” OR DE “Interviews” OR DE “Intake Interview” OR DE “Interview Schedules” OR DE “Psycho diagnostic Interview” OR DE “Grounded Theory” OR DE “Observation Methods” OR DE “Ethnography”0 OR DE “Discourse Analysis” OR DE “Content Analysis” OR DE “Phenomenology” OR DE “Philosophies” OR DE “Constructivism” OR DE “Hermeneutics” OR DE “Narratives” OR DE “Biography” OR DE “Life Review” OR DE “Storytelling” OR “qualitative research” OR “qualitative study” OR “qualitative method” OR “qualitative research” OR “qualitative study” OR “qualitative method”) AND (DE “Attitudes” OR DE “Knowledge (General)” OR DE “Psychology” OR DE “Management” OR psycholog* OR feeling OR attitude OR knowledge OR view OR perspective OR opinion OR experience OR image OR “self-concept” OR barriers OR management OR organization*) AND DE “Adolescent Health” OR “child” OR “adolescent” OR “young” OR “youth” OR “infant” OR “school age” OR “preschool” OR “child*” OR “teen*” AND DE “Sexual Abuse” OR DE “Incest” OR DE “Rape” OR DE “Sex Offenses” OR “sexual violence” OR “Child abuse” OR “sexual abuse” OR “sexual exploitation” OR “sexual assault” OR “molestation” OR “incest”
Cinahl	(MH “Qualitative Studies+”) OR (MH “Focus Groups”) OR (MH “Interviews+”) OR (MH “Narratives”) OR (MH “Observational Methods+”) OR (MH “Discourse Analysis”) OR (MH “Thematic Analysis”) OR (MH “Semantic Analysis”) OR (MH “Field Studies”) OR (MH “Audio recording”) OR (MH “Constant Comparative Method”) OR (MH “Content Analysis”) OR (MH “Field Notes”) OR (“qualitative research”) OR (“qualitative study”) OR (“qualitative method”)) AND ((MH “Attitude+”) OR (MH “Knowledge+”) OR (MH “Self-Concept+”) OR (MH “Psychology+”) OR (MH “Management+”) OR (“feeling”) OR (“attitude”) OR (“knowledge”) OR (“view”) OR (“perspective”) OR (“opinion”) OR (“experience”) OR (“image”) OR (“self-concept”) OR (“barriers”) OR (“management”) OR (“organization*”) OR (“psycholog*”) AND (MH “Child Abuse, Sexual”) OR (MH “Sexual Assault Examination”) OR (MH “Sibling Violence”) OR “sexual violence” OR (MH “Incest”) OR “incest” OR (MH “Rape”) OR “molestation” OR “sexual exploitation” OR (MH “Child Abuse Survivors”) AND “adolescent” OR (MH “Adolescent Health”) OR (MH “Adolescent Behavior”) OR (MH “Association of Child and Adolescent Psychiatric Nurses”) OR (MH “Adolescent Health Services”) OR (MH “Child+”) OR “child” OR (MH “Child Behavior+”) OR (MH “Infant+”) OR “infant”
SSCI	TS=(”case study” OR “constant comparative” OR “content analysis” OR “descriptive study” OR “discourse analysis” OR “ethnography” OR “ethnographic” OR “Focus group” OR “focus groups” OR “grounded theory” OR “interview*” OR “narrative*” OR “observation*” OR “qualitative method*” OR “qualitative research” OR “qualitative study” OR “thematic analysis” OR “semi-structured” OR “in depth”) AND TS=(“perception” OR “attitude” OR “feeling” OR “knowledge” OR “belief” OR “view” OR “perspective” OR “opinion” OR “experience” OR “image” OR “self-concept” OR “barrier*” OR “psycholog*” OR “management” OR “organization*”) AND TS=(“sexual abuse” OR “sexual exploitation” OR “sex abuse” OR “sexual violence” OR “rape” OR “sexual assault” OR “molestation”) AND TS=(“child” OR “teenager” OR “adolescent” OR “childhood” OR “infant” OR “school aged”)

### Study Selection

After the studies were collected and duplicates eliminated, two authors examined the titles and abstracts to assess their relevance (first screening). After reading all potentially pertinent articles in full, we selected only those articles that met our inclusion criteria (second screening). Table 2 describes the inclusion and exclusion criteria. Inclusion of the articles identified was debated at regular meetings of the research group, made up of specialists in qualitative research and of psychiatrists (specialized in treating children, adolescents, and adults). It was decided to keep all articles in which at least one paragraph of the results directly concerned the experience of disclosing sexual abuse to healthcare professionals, but only in a context of care. The experience of disclosure must have occurred before adulthood (age 18), but we included studies related to adults recounting this disclosure later on. We also decided not to include articles focused only on forensics and medicolegal aspects around the disclosure, for this issue, central in the experience of the disclosure and afterward, deserves its own systematic review of the qualitative literature, but is distanced from our research question because of its specialized participants and settings (police, criminal justice system).

**TABLE 2:** Inclusion and exclusion criteria.

	Inclusion criteria	Exclusion criteria
Design	Qualitative research	Quantitative and mixed studies
Article type	Peer-reviewed journal article	Reviews, commentaries, editorials, dissertations, non–peer-reviewed journal articles
Language	English	Other than English
Participants	-Survivors of child sexual abuse, adult or still minor at the time of the study, but both of whom had disclosed this abuse to a professional during childhood. -Parents of adolescent victims -Healthcare professionals	-Adult survivors who first disclosed child sexual abuse in adulthood. -Other professionals other than those in the care or education systems -Studies on sexual exploitation
Topic	Related to the lived experience of disclosure of child sexual abuse.	
Countries	All countries	

### Assessment of Study Quality

We used the Critical Appraisal Skills Program (CASP) to assess the quality of the articles included (24). CASP comprises 10 questions: two screening questions about the aims of the research and appropriate use of a qualitative methodology, and eight questions covering research design, sampling strategy, data collection, researcher’s reflexivity, ethical issues, data analysis, the findings, and the value of the research. Two authors (IB and EM) performed this assessment independently and then discussed the results within the research group until we reached agreement. Given the lack of consensus about the role and function of study quality assessment as part of systematic reviews, we did not exclude any study from the analysis based on our evaluation. Nonetheless, results are reliable when based on the studies with high methodological quality. We therefore performed a secondary sensitivity analysis by excluding from the synthesis studies in the lowest quartile of methodological quality.

The quality appraisal showed that the overall quality of the studies was high. Several papers failed to address the role of the researchers, that is, their own possible effects on the findings and/or interpretations (reflexivity item); others failed to report data collection and data analysis sufficiently.

### Extraction and Data Analysis

Two researchers (IB and TH) independently extracted data from the articles, that is, the first-order results (those of the study directly by the participants) and the second-order results (the authors’ interpretation and discussion of the results) in the form of a summary for each selected study. These data were analyzed independently by five researchers (IB, TH, AR-L, JS, and EJ). These analyses were discussed in detail at research group meetings. The thematic analysis followed the approach in five stages and enabled us to develop themes inductively, from the data. Stage 1 consisted of reading each summary over several times to obtain an overview of each study’s results. Stage 2 aimed to code each summary in cutting it up into descriptive units. The objective of stage 3 was to regroup/reorganize these units into categories by a process of synthesis, abstraction, and comparison, while stage 4 sought to identify the initial themes from the categories produced. In stage 5, we identified the studies’ recurrent themes and produced a coherently ordered table of themes and subthemes. The results’ high level of rigor is due to the simultaneous triangulation of data sources and analyses. NVivo 12 (QSR) software helped us to manage the data and facilitated the development of themes.

### Assessment of Confidence in the Findings

The CERQual (Confidence in the Evidence from Reviews of Qualitative research) GRADE approach (25) was used to assess confidence in the findings of the metasynthesis, following four key components: methodological limitations, relevance, coherence, and adequacy of the data. Assessment of these four components enabled us to reach a judgment about our overall confidence for each review finding, that is, each category in our results, rated as high, moderate, low, and very low, with “high confidence” being the starting assumption (26).

### Reflexivity

The research group made sure to work regularly on its reflexivity. The heterogeneity of the research group may thus have had a heuristic function: an intern in psychiatry, four child psychiatrists, one clinical psychologist, and a professor of somatic medicine. Some members of the group were sensitive to the question of the survivors’ outcome after the disclosure, and others to its negative consequences for them. The non-psychiatrist physician and the psychologist made it possible to avoid limiting the results to psychiatric perspectives alone.

## Results

**FIGURE 1:**
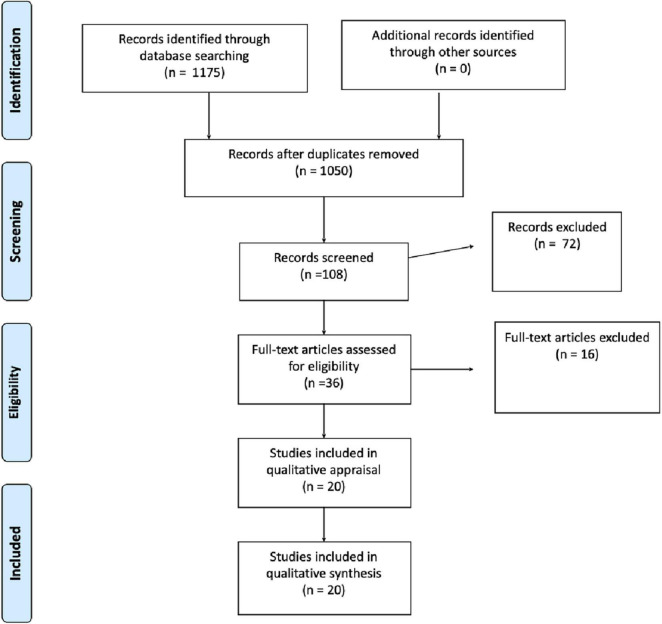
Flow chart records identified by database searches. The authors used the PRISMA guidelines to conduct their research (55).

Overall, we identified 1,175 initial references and finally selected 20 articles that met our inclusion criteria (Figure 1). The 20 studies we analyzed accounted for 2.2% of the initial 1,175 articles; this is the usual percentage in metasyntheses.

### Presentation of the Studies

The 20 studies came principally from English-speaking countries (Canada *N* = 7, United States *N* = 6, Australia *N* = 1, and Ireland *N* = 1) but also from the Netherlands (*N* = 1), Pakistan (*N* = 1), Norway (*N* = 1), and Sweden (*N* = 2). We found no French study. These studies included 20 articles, covering the data of 612 participants: 291 who were adults at the time of the study but were abused as children, 152 minors, 14 parents of adolescents, and 155 healthcare professionals: 23 school nurses, 19 pediatric dentists, 65 pediatric practitioners, 15 “other” healthcare professionals, 16 primary school teachers, and 17 public health nurses and physicians. No study specifically targeted professionals in the field of child psychiatry. No study among survivors specifically explored their experience of disclosing to healthcare professionals, rather the experience of disclosure in general whoever the person they disclosed to was.

Supplementary Table 2 reports the principal characteristics of these 20 studies.

### Quality and Confidence Evaluation

The quality of these articles, assessed according to CASP criteria, was globally good (Table 3). Only five studies met the criteria for reflexivity. Secondary analysis without the five studies with the lowest quality according to CASP did not change the results.

**TABLE 3:** Summary of the quality of the studies according to the Critical Appraisal Skills Program (CASP).

Principal criterion	Specific criteria (non-exhaustive list)	Quality assessment of the studies		
		Yes	Partially	No
Objectives	Explicit, relevant, important objectives	19	1	0
Method	Appropriate use of qualitative methods	19	1	0
Design	Design justified by the authors	17	3	0
Recruitment of participants	Recruitment described, appropriate, and justified by the authors	14	4	2
Data collection	Mode of collection clear, adequate, justified by the authors, data saturation discussed	13	6	1
Reflexivity of researchers	Researchers reflected about their own role and potential biases at different stages of the study	5	1	14
Ethical considerations	Approved by an ethics committee, details to participants	18	0	2
Data analysis	Specific description of the data analysis process, data sufficient to support the results	17	3	0
Results	Explicit, credible, discussed results	17	3	0
Value of the study	Contribution to existing knowledge, transferability, identification of new avenues of research	17	3	0

The CERQual assessment of the findings (Supplementary Table 1) showed “moderate confidence” (*N* = 19)—mainly due to «moderate methodological limitations» (*N* = 15)- and “low confidence” (*N* = 2) in the 21 categories. The six categories rated as “moderate concerns about adequacy” and five as “serious concerns about adequacy” were mainly based on studies offering thin data but no concerns about coherence or relevance. In many studies, disclosure was not the research question, hence the thinness of some data. In the “moderate confidence” categories, there were almost no concerns about coherence and relevance. The two “low confidence” categories are: *the negative effects of disclosure for the survivors reported by the healthcare professionals* (only two studies offering thin data); the *positive effect of disclosure experienced by the survivors just after disclosure (only one study).*

### Thematic Analysis

**FIGURE 2:**
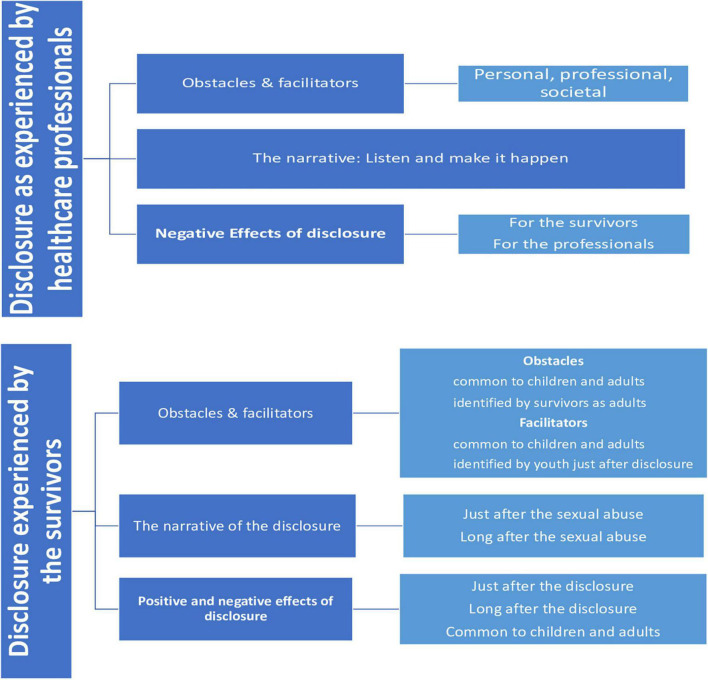
Presentation of the results.

The data analysis enabled us to uncover two themes that structured the experience around the disclosure of sexual abuse (Figure 2): (1) the disclosure as experienced by the professionals, and (2) the disclosure as experienced by the survivors. The quotations illustrating our results are assembled in a table (Supplementary Table 3).

#### Disclosure as Experienced by Healthcare Professionals

##### Obstacles and Facilitators

The healthcare professionals interviewed in these studies underlined both obstacles and facilitators in their ability to handle a CSA disclosure.

##### Obstacles

The obstacles were personal, professional, and societal.

##### Personal

Personal obstacles impeded the investigation and detection of CSA.

– Not the right person:

Some healthcare professionals considered that they were not the appropriate people to be told and manifested an “internal opposition” to these disclosures, especially in cases of intrafamily sexual abuse. Consequently, they would not try to elicit the disclosure (27, 28).

– The emotional weight:

Some professionals did not investigate—even avoided—the subject of sexual abuse if they felt uncertainty, solitude, discomfort, or fear of asking accusatory questions or of losing the alliance with the youth and/or his or her family, or of being able to tell the difference between well-being and maltreatment (27–31). Sexual abuse aroused strong feelings, opposition, and ambivalence among school nurses, both in thinking and talking about it (28). The authors underlined that these nurses had both knowledge and experience but that their emotional reactions were obstacles to detecting CSA and to taking adequate action about it (28).

– Repression:

According to the authors, the nurses studied avoided thinking about CSA—both unconsciously and consciously. They did not remember having been faced with disclosures of sexual abuse (28).

##### Professional

– Lack of training:

Lack of knowledge was invoked to explain uncertainty and the dilemma in both detecting and acting to deal with sexual abuse (28, 31). Some professionals felt discomfort discussing issues of sexuality and felt incompetent dealing with a spontaneous disclosure (27, 30).

– Workload and lack of time:

The lack of time resulted in a sense of powerlessness both to detect CSA and to support disclosure (27, 28).

##### Societal

– Shame:

Theories on shame may explain why the nurses in this study failed to address CSA in an attempt to avoid distressing feelings (28).

– Lack of services:

Some professionals mentioned the lack of appropriate referral services in case of CSA disclosure and their urgent need for it (27, 30).

– Societal weight:

Professionals point out the cultural barriers and social stigma about sexuality and CSA that impede its detection (29).

###### The Facilitators

##### Personal

– Certainty that disclosure is beneficial:

For some authors, it seemed essential that healthcare professionals be “certain of the beneficial effect of disclosure” to be able to investigate CSA (28). They have determined that the benefit-risk ratio of investigating sexual abuse is positive (27–30).

##### Professional

– Support and training:

Healthcare professionals investigated sexual abuse, when they identified alert signals and felt adequately supported and trained (27, 30).

– Have strategies:

Professionals used various strategies to get children to “open up” and help them to investigate.

##### Societal

– Professional responsibility:

Identifying CSA is seen as part of their professional responsibility (28–30).

##### The Narrative: Listen and Make It Happen

Healthcare professionals supported the disclosure narrative with different methods: by offering prevention messages, by asking specific or systematic questions during a routine investigation, by various forms of mediation (interview protocol, brochures, drawing, or writing) observing parents, the child, and the parent-child interactions, collecting collateral information, conducting home visits, and doing monitoring (27, 28, 30).

Some explained that they started the discussion with open questions, using simple terms, helping the youth to put what happened into words or to take part in the school’s regular sexual education and talks about puberty, and development of the body and sexual abuse with the child or the parents (27, 28). Some professionals insisted on the importance of building trusting relationships with pupils, partly through the pupils making contact themselves, partly through asking the pupil to come for an extra check-up (28).

Some authors moreover described the disclosure as an interpersonal and interactive process between the survivors and the healthcare professionals (28). Pediatric dentists identified two roles: being a supporter, if they had an alliance with the parents, or being a reporter (31).

##### Effects of Disclosure

###### Positive Aspects

####### For the Survivors

These studies found no positive aspects of disclosure for the survivors.

####### For the Healthcare Professionals

No study identified positive aspects of disclosure for the professionals.

####### Negative Aspects

######## For the Survivors

Participating healthcare professionals explained that these victims were often faced with problems in obtaining help and services: a lack of guidance, organization, and cooperation between different departments, a lack of means for keeping the child away from the abuser, the inadequacy of the laws about child protection. All of these resulted in a lack of confidence in both the support services and the families (29, 31).

######## For the Healthcare Professionals

Regardless of the circumstances of disclosure (actively investigated, spontaneous disclosure, CSA already suspected) healthcare professionals frequently reported negative feelings after the disclosure. They explained that they experienced difficult emotions, a sense of powerlessness, uncertainty; they felt vulnerable in these situation and feared burnout and consequences for themselves as much as for the child or adolescent (28, 29). They reported a lack of emotional support, structural tools, adequate time, and described a feeling of solitude (28, 30).

#### Disclosure Experienced by the Survivors

##### Obstacles and Facilitators

The participants reported that their experience of obstacles and facilitator had influenced their decision about whether or not to disclose CSA. Our results showed more obstacles to disclosure than factors facilitating disclosure. They distinguished obstacles and facilitators common to child or adolescent participants and adults, and other obstacles and facilitators identified only years after the abuse.

###### Obstacles to Disclosure

**Obstacles Common to Children and Adults**

– Shame and a feeling of responsibility:

Adults and minors described feelings of guilt, shame, and self-blame, which grew over time, sometimes throughout their entire lives (32–38).

– Fear of others’ reactions:

Some survivors underlined their anticipation of negative reactions from those they spoke to, especially the fear of not being believed (33, 35–39).

– Fear of consequences:

They were frightened both for themselves and for their families, about what would happen to them as well as to the perpetrator (32, 36, 37).

– Consequences of trauma:

The survivors described states of confusion, of ambivalence after the abuse (32, 34, 36, 40), or still partial or total denial of the abuse and what they called “forgetting,” especially during childhood, with “flashbacks” only in adulthood (35, 37, 41, 42). All these impede the process of disclosure (35, 37, 39, 43).

– Characteristics of and threats by the abuser:

All of the victims insisted on the abuser’s characteristics, which prevented disclosure—their sex, social status, and their relationship with the victims (32, 33, 36, 40), especially when they were a family member (33, 44). They reported the techniques abusers used to prevent their disclosure: threats (34, 35, 45), leading them to feel in danger (37), or on the other hand, tenderness, playing, or a gentle touch (32, 33, 35, 39, 45).

– Lack of words and knowledge about sexuality and sexual abuse or violence:

Some minors did not know what to say or how to say it; others described their persistence in trying to tell but not being able to say the words, or said that they did not perceive at that time that what they had experienced was sexual abuse (32, 36, 41). In the aftermath, adult survivors reported a lack of words and resources for disclosing this when they were children, which they associated with a lack of awareness about issues concerning their body, sexuality, and sexual abuse (34).

###### Obstacles Identified by Survivors as Adults, Years After Disclosure

In our studies, 99 of 291 adults did not tell anyone what happened to them as children, three did not remember their exact age at disclosure.

– Being a boy:

Boys feared how society would see them (potential future abuser, homosexual) and the resulting stigma of being labeled as homosexual (37, 42, 46).

– Lack of needed knowledge:

Some did not know anything about their body, boundaries, sexuality, and CSA (39).

– Family and societal environment:

The adult survivors described family dysfunctions that could impede disclosure, such as a rigid, patriarchal family structure, social isolation, and poor intrafamily communication (35, 44).

Feeling unprotected by the adults around them: some adults participants explained that, as children, their failed attempts led them to feel unprotected by the adults surrounding them (39, 42). They could also feel that nobody would listen to them (42).

– Societal influence:

Patriarchal societies for male survivors (40, 46), the fear of being considered insane in their society (35), and societies where it was forbidden/taboo to talk about sex (46)—all of these prevented disclosure by survivors.

###### Facilitators of Disclosure

**Facilitators Common to Children and Adults**

– Characteristics of the person to whom the disclosure is made:

A reassuring, supportive, safe, adequately close professional, who listens and takes an interest in the survivor promotes disclosure (37, 39, 41, 45).

– Choice of a trustworthy person:

Some victims also explained the need for a trusting relationship with the person before they are able to tell them. They underlined the importance of choosing their confidant (34, 41, 42, 45).

– Societal influence:

They described how the experience of disclosure was influenced by contextual elements such as meeting other victims (42, 45, 46) or media use (42, 46).

– Internal/personal motivations:

Some victims said they told someone to put an end to the sexual abuse, to protect themselves and other victims (33, 34, 39, 42) and to escape their solitude (28, 38, 42), but also that they needed to understand what was happening (33, 37).

###### The Facilitators Identified by Youth, Just After the Disclosure

– Opportunity to tell:

Many young people noted that absent the particular set of circumstances at the time of disclosure told (an argument, watching a television program about abuse)— they would not have told anyone at all or would not have told for some time (41).

– The right moment:

Some young participants expressed the importance of choosing the circumstances of disclosure, in a familiar and safe environment, and to feel tacit permission to talk about it (45).

##### The Narrative of the Disclosure

###### Just After the Sexual Abuse

Some minors communicated the complexities, resulting challenges, and personal embarrassments that often accompanied these disclosures (36). They could no longer keep this dark secret and decided to talk about it “to another person,” often a family member. Some described having disclosed this directly to someone they were talking to, using vague or unspecific language or in the form of questions (34, 45). Children and adolescents described two types of disclosure: a controlled, progressive narrative, spaced over time to prepare the other person to learn the secret (41, 45) and uncontrolled disclosure, described by the authors as unplanned and touched off unexpectedly (41, 45). These young survivors describe the multiple reactions of the people hearing this secret—shocked, sad, angry, or blaming (36).

###### Long After the Sexual Abuse

Adults principally reported either the experience of their first disclosure or experience of non-disclosure. The first disclosure was described as an important moment in their lives (39, 46). They also described the different types of disclosure: intentional planned disclosure, disclosure elicited by the professional (investigative/survey interviews, play…) sometimes induced by a detail, accidental disclosure, “intentionally guarded” disclosure, disclosure triggered by a flashback at specific moments, and finally indirect disclosure, in the form of allusion, or changed behavior, such as by adolescent acting out (33, 37, 38, 42, 43). The disclosure was described as a progressive, dynamic process lasting through multiple narrations of disclosure to different confidants throughout their lives (33, 37, 42). They had always experienced the moments of non-disclosure, when they had failed to tell someone, negatively and were left with feelings of solitude, regret, and ambivalence years later (42).

##### The Effects of Disclosure

All of these studies underlined the emotional, organizational, and structural implications that followed disclosure for the participants. The participants described: (1) the positive aspects and (2) negative aspects of the disclosure, which could be identified just after the disclosure, or years later, or be similar for children and adults.

###### Positive Aspects

###### Just After the Disclosure

Among the children and adolescents, only those who received counseling interventions about sexual abuse from trained counselors (36) were able to take the same distance as adults to the effects of disclosure on themselves and were thus able to develop a future.

###### Long After the Disclosure

Some adult survivors talking about the disclosure experience years later described this first telling about their abuse as a release; they spoke of “bringing it up,” “opening up,” and “getting it out” (28). The experience of disclosure could be described as extremely beneficial and exclusively positive (39, 42, 46). The disclosure first allowed them to be believed and heard, brought them knowledge that the abuse was not their fault (42), and resulted in feeling supported, listened to, emotionally respected, and treated compassionately in a non-therapeutic context (42). Moreover, some underlined the crucial importance of protective measures such as legal placement, changes of school, eviction of the abuser from the household, and referral to specific types of care and specific legal bodies (42).

###### Common to Children and Adults

The survivors described feeling “normal,” proud, and not guilty, which also enabled them to feel reassured and helped and to find meaning in their story (33, 34, 36). Moreover, associations with those around them were preserved when the survivors felt that they were listened to and believed (34, 42).

###### Negative Aspects

###### Just After the Disclosure

Some minors reported their disclosure experience left them emotionally confused, with a feeling of loss of control and instability both at the time and in the aftermath of the disclosure (34, 36). Those who received a counseling intervention described all that they had lost: loss of trust, loss of relationships with the perpetrators or other family members, loss of friends as a results of moving (36). Some minors were mocked at school and reported negative effects in their interpersonal peer relationships and also in their family (34). They saw their world split into categories: those who have been abused and those who have not, those who know about them and those who do not (34, 36).

Just after the disclosure, some minors described important and abrupt changes in their lives after legal decisions that they experienced as a violation of their personal/private life (34, 36). They also described the consequences linked to investigations that were “long, hard, brutal, and seemed like never ending.” These generated feelings of frustration among children and adolescents, above and beyond the examinations to collect physical evidence (36).

###### Long After the Disclosure

The adults remembered the reactions of people who received the disclosure, reactions that they judged unsupportive and unsatisfactory and that left them feeling both abandoned and responsible for the abuse (37, 42). Some explained that they had then begun to question and even doubt the abuse, even to the point of denying it (37).

###### Common to Children and Adults

All the survivors described painful experiences after the disclosure. They felt fear, embarrassment, guilt, pride, sadness, shame, anger, powerlessness, vulnerability, and anger at betrayal; these sometimes led them to ER visits (34, 36, 37, 43). They very often described inappropriate responses by families and others around them after the disclosure. Some were threatened by their families, who sometimes tried to hide the secret (34, 37, 41, 42). Some experienced forms of disclosure-related trauma: either because nothing changed for the better or because they were not believed or supported (34, 37) or were asked to choose between the perpetuator and the other parent (37). Most participants explained that the survivors often faced problems in obtaining assistance, leading to a lack of confidence in their support services and in their families (34, 46).

## Discussion

This metasynthesis has crossed the lived experience of CSA disclosure from the viewpoints of survivors at two stages after disclosure—shortly afterward (adolescent participants) and many years later (adult participants)—and the perspectives of healthcare professionals. It is important to mention that no qualitative study exploring the views of survivors has focused on the issue of disclosure to healthcare professionals. Further research to focus on this situation is therefore needed to explore in depth this important and not uncommon issue in daily clinical practice. Similarly, no study has explored the process of disclosure in the field of child psychiatry. Future research specifically in this field is necessary and especially important in light of the epidemiologic data available (47).

Some aspects of our results have previously been described in the literature: on the one hand, disclosure as a means of entering into a phase of repair and recovery, attenuation of the survivors’ symptoms and distress (9, 10); on the other hand, the obstacles that keep professionals from investigating sexual abuse, such as fear of losing their therapeutic alliance, the difficulty of raising a topic judged taboo, lack of time, as well as feelings of discomfort, fear, and anxiety (48). Other studies have already described professionals’ experience of uncertainty and solitude after their patients’ disclosure of the CSA (49).

The diachronic approach to the survivors’ experience of disclosure appears on the other hand to be an original result of our metasynthesis. Our findings suggest a change over time in the understanding of this experience: relief and release were seen only among the adult participants, at a distance from the disclosure, and in participating adolescents who had received specific interventions and specialized support. This result perfectly illustrates the need for specific support for survivors in the aftermath of disclosure (36). The earliness of the disclosure is a known protective factor against psychological distress in the short, medium, and long terms (50). The question of temporality must therefore be considered in looking at the experience of disclosing sexual abuse. Until now, no study has investigated this experience longitudinally, that is, examined its changes with time. Exploring the effects of disclosure in the short, medium, and long term appears crucial to be able to understand what survivors who disclosed their abuse in childhood have lived—and continue to live. Further research with prospective design should therefore investigate the evolution of the perception of the disclosure and its outcome with time as our results suggest that awareness of these changes could affect healthcare professionals’ attitudes toward CSA disclosure. That is, beyond the obstacles and the negative aspects already known in the literature for survivors before and after disclosure (21), our results show that healthcare professionals undergo an experience that mirrors that of survivors, in particular those survivors interviewed shortly after the disclosure: experience of solitude, uncertainty, discomfort, and fear, but also of the negative, even traumatic experiences of disclosure. This is closely akin to the concepts of “vicarious trauma” (51) and “burden sharing,” already described in the context of CSA disclosure within families (12) or with peers (52). The experiences of disclosures with negative effects, both for survivors and healthcare professionals, can have an iatrogenic effect on the disclosure process, obstructing both the survivor’s voice and the professionals’ investigation. As a corollary, we think that application of the principle of patient experts (53), by involving now-adult survivors, at a distance from their experience of disclosure, in the initial and continued training of the healthcare professionals concerned would make it easier for professionals to investigate and support the disclosure process in their young patients by having integrated its long-term effects.

### Clinical Implications

Practical clinical implications can be drawn from the results of this metasynthesis.

1. “It takes time”: healthcare professionals need to address to the survivors and themselves the issues of temporality, that is the process of disclosure takes time but also that positive outcome of the disclosure will occur later in life.
2. “We need support to support”: To overcome the feelings of solitude, uncertainty, discomfort, and fear related with the experience of disclosure, healthcare professionals need to have support groups, pluridisciplinary teams and clinical supervisions.
3. “Eliciting disclosure is not enough”: Not only, healthcare professionals—especially the one working closely with children- need to be proactive to detect CSA and elicit disclosure, they also need to be supportive and to provide counseling to their patient after the disclosure to ensure that he or she will benefit from having disclosed.

### Strengths and Limitations

This review incorporates the points of view about the disclosure of CSA of 291 adult survivors, 152 survivors still minors at the time of the study, and 155 healthcare professionals. The method that we applied is rigorous (54) and meets the ENTREQ guidelines’ criteria of rigor and quality. We analyzed 20 articles, all published in peer-reviewed journals and most of them of good methodological quality.

Nonetheless, certain aspects of this metasynthesis limit the generalization of its conclusions. A qualitative metasynthesis collects only partial data from the participants and the interpretations of the researchers, which are the data given in the initial articles. Moreover, although the review assembled articles from diverse cultural areas, English-speaking countries are overrepresented as we restricted our selection to articles in that language.

Several articles included in our study most probably derive from the same original studies (38, 40–43, 46). Although they explore different aspects of the experience of disclosure to a health professional, there is a risk that they carry more weight than others in our results.

Moreover, because research among adults involves fewer ethical and regulatory constraints, this review includes more studies including adults (*N* = 291) than those who are still minors (*N* = 152). We have sought to prevent, insofar as possible, any influence of this overrepresentation on our results. Nonetheless, more studies exploring the experience of disclosure among child and adolescent survivors seem necessary to confirm the points raised in our discussion.

Moreover, there are two important limitations concerning the broad scope of this metasynthesis and the lack of qualitative study specifically exploring the experience of children and adolescents disclosing CSA to health care professionals. Qualitative literature on CSA disclosure to healthcare professionals is scarce and led us to a systematic review gathering different professionals and healthcare contexts. Further qualitative research focusing on disclosing within the context of care is necessary, especially from the point of view of survivors. In this metasynthesis, in order to avoid being confined to the sole perspectives of professionals, we decided to include the studies exploring survivors’ experience of CSA disclosure, whomever the person they disclosed to was.

Finally, this decontextualization prevents us from adequately taking into account the cultural factors, despite their evident importance. Specific studies, anthropological work, and transcultural investigations would make it possible to approach these aspects.

## Conclusion

This metasynthesis has enabled us to propose new avenues for improving the process of the disclosure of CSA to healthcare professionals: an experience that mirrors that of professionals and of minor survivors, and a more positive viewpoint further away from the process in survivors who are now adults. This has enabled us to identify new research perspectives such as the specific exploration of the stakes of disclosure in the field child psychiatry, examination of the experience of the survivor-professional dyad through qualitative studies that cross their perspectives, and to set up prospective studies to better describe the effects of disclosure on professionals over time. Finally, this metasynthesis allowed us to identify prospects for educating professionals by involving adult survivors, in the manner of patient-experts, in the initial and continued training of healthcare professionals.

## Author Contributions

JS, IB, AR-L, TH, LV, and EM conceived and designed the experiments and final approval. IB, EJ, and TH conducted the literature review. IB, TH, EJ, and EM analyzed the data. JS, IB, and EM wrote the manuscript. AR-L wrote the analysis. JS and EM wrote the introduction, analysis, and discussion. All authors contributed to the article and approved the submitted version.

## Conflict of Interest

The authors declare that the research was conducted in the absence of any commercial or financial relationships that could be construed as a potential conflict of interest.

## Publisher’s Note

All claims expressed in this article are solely those of the authors and do not necessarily represent those of their affiliated organizations, or those of the publisher, the editors and the reviewers. Any product that may be evaluated in this article, or claim that may be made by its manufacturer, is not guaranteed or endorsed by the publisher.
