# Supplementary material for: Disclosing Child Sexual Abuse to a Health Professional: A Metasynthesis
Source: Front Psychiatry. 2022 Jun 3;13:788123. doi: 10.3389/fpsyt.2022.788123 (PMC9211373; doi:10.3389/fpsyt.2022.788123)
Supplement: Supplementary file 1 [file Table_1.DOCX]

**Supplementary Material 1. CERQual of Review Findings**

| **Summary of review findings** | **Studies contributing to the review findings** | **Methodological limitations** | **Coherence** | **Adequacy** | **Relevance** | **Assessment of the confidence in the evidence** | **Explanation of CERQUal assessment** |
| --- | --- | --- | --- | --- | --- | --- | --- |
| 1. **Disclosure as experienced by healthcare professionals** | | | | | | | |
| - 1. **Obstacles and facilitators** | | | | | | | |
| - - 1. Obstacles | | | | | | | |
| 1.1.1.1.  Personal | 27, 28, 29, 30, 31 | Moderate methodological limitations (2 studies with minor methodological limitations (no reflexivity), three with moderate methodological limitations (unclear recruitment and sampling strategy and unclear data analysis)) | No or very minor concerns about coherence | No or very minor concerns about adequacy | No or very minor concerns about relevance (5 studies across three continents, with direct relevance) | Moderate confidence | Minor concerns regarding coherence, adequacy and relevance, moderate concerns regarding methodological limitations |
| 1.1.1.2.  Professional | 27, 28, 30, 31 | Moderate methodological limitations (1 study with minor methodological limitations (no reflexivity); three with moderate methodological limitations (unclear recruitment and sampling strategy and unclear data analysis)) | No or very minor concerns about coherence | No or very minor concerns about adequacy | Moderate concerns about relevance (4 studies across two continents but only in rich countries (USA, Sweden and Netherlands)) | Moderate confidence | Minor concerns regarding coherence and adequacy, moderate concerns regarding methodological limitations and relevance |
| 1.1.1.3.  Societal | 27, 28, 29, 30 | Moderate methodological limitations (1 study with minor methodological limitations (no reflexivity); three with moderate methodological limitations (unclear recruitment and sampling strategy and unclear data analysis)) | No or very minor concerns about coherence | Moderate concerns about adequacy (studies offering thin data) | No or very minor concerns about relevance (4 studies across three continents, with direct relevance) | Moderate confidence | Minor concerns regarding coherence, adequacy and relevance, moderate concerns regarding methodological limitations |
| - - 1. The facilitators | | | | | | | |
| 1.1.2.1.  Personal | 27, 28, 30 | Moderate methodological limitations (1 study with minor methodological limitations (no reflexivity); 2 studies with moderate methodological limitations (unclear recruitment and sampling strategy and unclear data analysis, no reflexivity)) | No or very minor concerns about coherence | Moderate concerns about adequacy (only 3 studies) | No or very minor concerns about relevance (3 studies across three continents, with direct relevance) | Moderate confidence | Minor concerns regarding coherence and relevance, moderate concerns regarding methodological limitations and adequacy |
| 1.1.2.2.  Professional | 27, 28, 30 | Moderate methodological limitations (1 study with minor methodological limitations (no reflexivity); 2 studies with moderate methodological limitations (unclear recruitment and sampling strategy and unclear data analysis, no reflexivity)) | No or very minor concerns about coherence | Moderate concerns about adequacy (only 3 studies) | No or very minor concerns about relevance (3 studies across three continents, with direct relevance) | Moderate confidence | Minor concerns regarding coherence and relevance, moderate concerns regarding methodological limitations and adequacy |
| 1.1.2.3.  Societal | 28, 29, 30 | Moderate methodological limitations (3 studies with moderate methodological limitations (unclear recruitment and sampling strategy, unclear data analysis, no reflexivity)) | No or very minor concerns about coherence | Moderate concerns about adequacy (only 3 studies) | No or very minor concerns about relevance (3 studies across three continents, with direct relevance) | Moderate confidence | Minor concerns regarding coherence and relevance, moderate concerns regarding methodological limitations and adequacy |
| - 1. **The narrative: Listen and make it happen** | | | | | | | |
|  | 27, 28, 30, 31 | Moderate methodological limitations (1 study with minor methodological limitations (no reflexivity); three with moderate methodological limitations (unclear recruitment and sampling strategy and unclear data analysis)) | No or very minor concerns about coherence | No or very minor concerns about adequacy | Moderate concerns about relevance (4 studies across two continents but only in rich countries (USA, Sweden and Netherlands)) | Moderate confidence | Minor concerns regarding coherence and adequacy, moderate concerns regarding methodological limitations and relevance |
| - 1. **Effects of disclosure** | | | | | | | |
| - - 1. Positive aspects None | | | | | | | |
| - - 1. Negative aspects | | | | | | | |
| For the survivors | 29, 31 | Moderate methodological limitations (2 studies with moderate methodological limitations (unclear recruitment and sampling strategy, unclear data analysis, and no reflexivity)) | No or very minor concerns about coherence | Serious concerns about adequacy (only 2 studies, studies offering thin data) | No or very minor concerns about relevance (2 studies across two continents (Asia and Europe) | Low confidence | Minor concerns regarding coherence and relevance, moderate concerns regarding methodological limitations and serious concerns regarding adequacy |
| For the healthcare professionals | 28, 29, 30 | Moderate methodological limitations (3 studies with moderate methodological limitations (unclear recruitment and sampling strategy, unclear data analysis and no reflexivity)) | No or very minor concerns about coherence | Moderate concerns about adequacy (only 3 studies) | No or very minor concerns about relevance (3 studies across three continents, with direct relevance) | Moderate confidence | Minor concerns regarding coherence and relevance, moderate concerns regarding methodological limitations and adequacy |
| 1. **Disclosure experienced by the survivors** | | | | | | | |
| - 1. **Obstacles and facilitators** | | | | | | | |
| - - 1. **Obstacles to disclosure** | | | | | | | |
| Obstacles common to children and adults | 32, 33, 34, 35, 36, 37, 38, 39, 40, 41, 42, 43, 44, 45 | Moderate methodological limitations (4 studies with no or minor methodological limitations (no reflexivity for 1); 10 studies with moderate methodological limitations (unclear data collection; unclear recruitment and sampling strategy; unclear data analysis, and 2 studies without ethical issue item)) | No or very minor concerns about coherence | No or very minor concerns about adequacy (14 studies, all with rich data) | No or very minor concerns about relevance (14 studies across three continents, with direct relevance) | Moderate confidence | Minor concerns regarding coherence, adequacy and relevance, moderate concerns regarding methodological limitations |
| Obstacles identified by survivors as adults, years after disclosure | 35, 37, 39, 40, 42, 44, 46 | Moderate methodological limitations (3 studies with no or minor methodological limitations (no reflexivity for 2); 4 studies with moderate methodological limitations (unclear method and research design; unclear data analysis, unclear recruitment and sampling strategy)) | No or very minor concerns about coherence | No or very minor concerns about adequacy (7 studies, all with rich data) | No or very minor concerns about relevance (7 studies across three continents, with direct relevance) | Moderate confidence | Minor concerns regarding coherence, adequacy and relevance, moderate concerns regarding methodological limitations |
| - - 1. **Facilitators of disclosure** | | | | | | | |
| Facilitators common to children and adults | 33, 34, 37, 38, 39, 41, 42, 45, 46 | Moderate methodological limitations (5 studies with no or minor methodological limitations (no reflexivity for 2); 4 studies with moderate methodological limitations (unclear data collection; unclear recruitment and sampling strategy; unclear data analysis and unclear research design)) | No or very minor concerns about coherence | No or very minor concerns about adequacy (9 studies, all with rich data) | No or very minor concerns about relevance (9 studies across three continents, with direct relevance) | Moderate confidence | Minor concerns regarding coherence, adequacy and relevance, moderate concerns regarding methodological limitations |
| The facilitators identified by youth, just after the disclosure | 41, 45 | Minor methodological limitations (1 study with minor methodological limitations (no reflexivity and unclear data collection)) | No or very minor concerns about coherence | Serious concerns about adequacy (only 2 studies) | No or very minor concerns about relevance (2 studies across two continents, with direct relevance) | Moderate confidence | Minor concerns regarding methodological limitations, coherence and relevance, serious concerns regarding adequacy |
| - 1. **The narrative of the disclosure** | | | | | | | |
| Just after the sexual abuse | 34, 36, 41, 45 | Minor methodological limitations (1 study with minor methodological limitations (no reflexivity); 3 studies with moderate methodological limitations (unclear recruitment and sampling strategy; data collection)) | No or very minor concerns about coherence | Moderate concerns about adequacy (only 4 studies) | Moderate concerns about relevance (4 studies across two continents, with direct relevance, but three from USA) | Moderate confidence | Minor concerns regarding methodological limitations and coherence, moderate concerns regarding adequacy and relevance |
| Long after the sexual abuse | 33, 37, 38, 39, 42, 43, 46 | Moderate methodological limitations (4 studies with no or minor methodological limitations (no reflexivity for 2); 3 studies with moderate methodological limitations unclear data collection and unclear data analysis)) | No or very minor concerns about coherence | No or very minor concerns about adequacy (7 studies, all with rich data) | No or very minor concerns about relevance (7 studies across 3 continents, with direct relevance) | Moderate confidence | Minor concerns regarding coherence, adequacy and relevance, moderate concerns regarding methodological limitations |
| - 1. **The effects of disclosure** | | | | | | | |
| - - 1. Positive aspects | | | | | | | |
| Just after the disclosure | 36 | Moderate methodological limitations (1 study with unclear recruitment and sampling strategy, unclear data collection) | No or very minor concerns about coherence | Serious concerns about adequacy (only 1 study) | Moderate concerns about relevance (partial relevance as the studies were from only one setting) | Low confidence | Minor concerns regarding coherence, moderate concerns regarding methodological limitations and relevance, serious concerns regarding adequacy |
| Long after the disclosure | 33, 39, 42, 46 | Moderate methodological limitations (2 studies without reflexivity item; 1 study with unclear method and research design, unclear data collection and 1 study with unclear data analysis) | No or very minor concerns about coherence | No or very minor concerns about adequacy | No or very minor concerns about relevance (4 studies across two continents, with direct relevance) | Moderate confidence | Minor concerns regarding coherence, adequacy and relevance, moderate concerns regarding methodological limitations |
| Common to children and adults | 33, 34, 36, 42 | Moderate methodological limitations (1 study without reflexivity item; 2 with unclear data collection; 2 with unclear recruitment and sampling strategy; 1 with unclear data analysis) | No or very minor concerns about coherence | No or very minor concerns about adequacy | Moderate concerns about relevance (4 studies across only 1 continent, North America) | Moderate confidence | Minor concerns regarding coherence and adequacy, moderate concerns regarding methodological limitations and relevance |
| - - 1. Negative aspects | | | | | | | |
| Just after the disclosure | 34, 36 | Moderate methodological limitations (2 studies with unclear recruitment and sampling strategy and unclear data collection) | No or very minor concerns about coherence | Serious concerns about adequacy (only 2 studies) | Moderate concerns about relevance (2 studies from only one setting) | Moderate confidence | Minor concerns regarding coherence, moderate concerns regarding methodological limitations and relevance, serious concerns regarding adequacy |
| Long after the disclosure | 37, 42 | Minor methodological limitations (1 study without reflexivity item and one with unclear data analysis) | No or very minor concerns about coherence | Serious concerns about adequacy (only 2 studies) | No or very minor concerns about relevance (2 studies across two continents, with direct relevance) | Moderate confidence | Minor concerns regarding methodological limitations, coherence and relevance, serious concerns regarding adequacy |
| Common to children and adults | 34, 36, 37, 41, 42, 43 | Moderate methodological limitations (3 studies with minor methodological limitations (no reflexivity for 2); 3 with moderate methodological limitations (1 without ethical issue item; unclear recruitment and sampling strategy unclear data collection and unclear data analysis)) | No or very minor concerns about coherence | No or very minor concerns about adequacy (6 studies with rich data) | No or very minor concerns about relevance (6 studies across three continents, with direct relevance) | Moderate confidence | Minor concerns regarding coherence, adequacy and relevance, moderate concerns regarding methodological limitations |
