# Supplementary material for: Disclosing Child Sexual Abuse to a Health Professional: A Metasynthesis
Source: Front Psychiatry. 2022 Jun 3;13:788123. doi: 10.3389/fpsyt.2022.788123 (PMC9211373; doi:10.3389/fpsyt.2022.788123)
Supplement: Supplementary file 2 [file Table_2.DOCX]

Supplementary Material 2. Main Characteristics of the included studies

| **Authors** | **Year** | **Objective(s)** | **Country** | **Setting** | **Participants** | **Data collection** | **Method of analysis** |
| --- | --- | --- | --- | --- | --- | --- | --- |
| Alaggia | 2004 | To identify influences that inhibit or promote children’s disclosure of child sexual abuse | Canada | Clinical and non-clinical sample (through community agencies, two university campuses and word of mouth) | **Adult survivors**  N=24 (aged between 18 and 65) | SSI | Thematic analysis |
| Alaggia | 2005 | To explore dynamics that impede or promote disclosure of child sexual abuse. | Canada | Non-clinical sample (large multicultural Canadian city) | **Adult survivors**  N=30 (aged between 18 and 65; 19 women and 11 men) | SSI | Thematic analysis |
| Alaggia & Kirshenbaum | 2005 | To identify a broad range of factors, including family dynamics, that contribute to or hinder a child’s ability to disclose sexual abuse. | Canada | Clinical and non-clinical sample (through community agencies, two university campuses and word of mouth) | **Adult survivors**  N=20 (12 women and 8 men) | SSI | Thematic analysis |
| Alaggia & Millington | 2008 | To understand the lived experience of men sexually abused as boys in their childhood, and what life is like for them as sexual abuse survivors in adulthood. | Canada | Clinical population (Through social service agencies) | **Adult survivors**  N=14 men (aged between 28 and 65) | SSI | Thematic analysis |
| Brattfjell | 2019 | To explore exposed own experiences of steps towards final disclosure. | Norway | Norwegian Sexual Abuse Support Centers | **Adult survivors**  N=23 (22 women, 1 man; aged between 29 and more than 50) | Survey | Interpretative phenomenological analysis |
| Collin-Vézina et al. | 2015 | To provide a preliminary mapping of the barriers to CSA disclosures through an ecological systemic lens | Canada | Community-based sexual assault or adult counselling/mental health services | **Adult survivors**  N=67 (51 female, 16 male; aged between 19 and 69) | SSI | Grounded theory method |
| Draucker & Martsolf | 2008 | To explain how survivors of childhood sexual abuse tell others about their abuse experiences | USA | Non-clinical sample | **Adult survivors**  N=74 (40 women and 34 men, aged between 18 and 62) | SSI | Grounded theory method |
| Engh Kraft et al. | 2017 | to explore the ability of the school nurses to detect and support sexually abused children. | Sweden | Schools | **Health professional**  N=23 (all women; aged between 46 and 67) | Focus Group (FG) | Thematic analysis |
| Foster and Hagedorn | 2014 | To explore trauma narratives written by children as part of a counseling intervention | USA | Large, urban child advocacy center that counsels child victims of sexual abuse | **Minor survivors**  N=21 (18 girls and 3 boys; aged between 6 et 17) | Trauma narratives | Narrative analysis |
| Gagnier et al. | 2017 | To explores the journey of obtaining services for adult male survivors of child sexual abuse | Canada | Organizations specializing in sexual abuse and mental health organizations | **Adult survivors**  N= 17 (all men; aged between 19 and 67) | SSI | Phenomenological approach |
| Gagnier & Collin-Vezina | 2016 | To explore the diversity in the disclosure process of male survivors of child sexual abuse. | Canada | Through community and mental health organizations | **Adult survivors**  N= 17 (all men; aged between 19 and 67) | SSI | Phenomenological approach |
| Hassan et al. | 2015 | To explore the young victim's own stories of disclosure of child sexual abuse and is an exploratory case study using reported assault histories of victims of CSA between the ages of 6 and 14, taken in the immediate wake of the assault. | USA | Emergency department (hospital) | **Minor survivors**  N=37 (33 girls and 4 boys; aged between 6 and 14) | SSI | Thematic analysis |
| Hunter | 2011 | to develop a fuller understanding of the process of disclosure of child sexual abuse. | Australia | Non-clinical sample (via press releases and interviews on local radio) | **Adult survivors**  N= 22 (9 men and 13 women, aged between 25 and 70) | SSI | Rosenthal et Fischer-Rosenthal method |
| Kvis et al. | 2014 | To examine the factors that lead specialists in pediatric dentistry to suspect child abuse or neglect and the considerations that influence the decision to report these suspicions to social services. | Sweden | Pediatric dentistry centers | **Pediatric dentistry**  N=19 | FG | thematic analysis |
| Leder et al. | 1999 | To describe factors that prompt pediatric practitioners to suspect child sexual abuse, the barriers to inquiry, and the approach to man- agement of cases of possible abuse. | USA | Maternal and Child Health Bureau-sponsored Collaborative office rounds groups | **Pediatric practitioners**  N=65 | FG | thematic analysis |
| Maul et al. | 2019 | To identify shared experiences of HCP and the challenges they face when managing cases of child abuse; to explore social and cultural factors in Pakistani society that may affect the way in which children who have been abused present  and how this affects their treatment; and to understand the child abuse training that HCP have already received and identify any further requirements. | Pakistan | A large, private hospital within Pakistan. | **Healthcare professionals**  N= 15 | SSI | Inductive analysis |
| Mc Elvaney et al. | 2012 | To explore how chil- dren tell of their experiences of child sexual abuse from the perspectives of young people and their parents. | Irland | Child sexual abuse assessment and therapy service, based in a children’s hospital in a large city | **Minor survivors**  N= 22 (16 girls and 6 boys, aged between 8 and 18)  &  **Parents**  N=14 | SSI | Grounded theory method |
| Petronio et al. | 1996 | To examine child and adolescent disclosures of sexual abuse. | USA | Not-for-profit agency | **Minor survivors**  N= 38 (32 girls and 6 boys, aged between 8 and 18) | SSI | Thematic analysis |
| Schols et al. | 2013 | To investigate Dutch frontline workers’ child abuse detection and reporting behaviors. | Netherlands | Two Dutch local health services organization | **Professionals**  N=33  (16 primary school teachers and 17 public health nurses and physicians) | FG | Thematic analysis |
| Staller & Nelson-Gardell | 2005 | To enhance understanding of the sexual abuse disclosure process from the perspective of preteen and teenage survivors. To reconsider prominent models of the disclosure process in light of our findings. | USA | Non-clinical sample | **Minor survivors**  N= 34 (all girls; aged between 10 and 18) | FG | Thematic analysis |
