# Supplementary material for: Disclosing Child Sexual Abuse to a Health Professional: A Metasynthesis
Source: Front Psychiatry. 2022 Jun 3;13:788123. doi: 10.3389/fpsyt.2022.788123 (PMC9211373; doi:10.3389/fpsyt.2022.788123)
Supplement: Supplementary file 3 [file Table_3.DOCX]

**Supplementary Material 3. Quotations from Patients, PCPs and Authors of primary studies to illustrate each theme**

| 1. **1. Disclosure as experienced by healthcare professionals** | |
| --- | --- |
| - 1. **Obstacles and facilitators** | |
| - - 1. **Obstacles** | |
| - - - 1. Personal | **HCP:** ‘‘. . . we just can’t save everyone. You can’t get involved in every case.’’ (28)  **HCP:** “There’s the risk that if you suspect it [sexual abuse] and start evaluating it . . . you can really even make it worse for the family.” (27)  **HCP:** ‘‘Do we really want to know this information and then [have to] deal with it?” (27)  **HCP:** “I have to admit honestly that in this case I probably have been very naïve. I never thought of child sexual abuse. I still think about this case very often. That I did not think of sexual abuse. I just thought that it was a badly raised child and it did not cross my mind that there could be something really wrong”. (30) |
| 1.1.1.2. Professional | **HCP:** “It [the decision to inquire about possible sexual abuse] is a major decision and nothing in residency prepared me for the decision when I first started practice.” (27)  **A^HCP^:** ‘‘Previous studies support these findings. A recente nationwide survey of pediatric residency program directors found that child abuse, particularly sexual abuse, is an area that is not covered adequately during training” (27) |
| 1.1.1.3. Societal | **HCP:** ‘‘It is such a taboo area. It is so awful if it is true.’’ (28) |
| - - 1. **The facilitators** | |
| - - - 1. Personal | **HCP**: “It happens often to me that things happen in a flash and then I do not know what I have noticed specifically, but I do have this gut-feeling that something is wrong”. (30) |
| - - - 1. Professional | **HCP:** ‘‘She was a quiet and timid girl who showed no signs. It was just that she told me . . . I don’t know what other opportunity she would have chosen.’’ (28)  **HCP:** ‘‘(I) told the girls that this was great but that we would deal with it from now on.’’ (28)  **HCP**: “But then it is about not wanting to break off the relationship with parents. You want to do it together with the parents. But sometimes this is not possible anymore, but when is this point reached?” (30) |
| - - - 1. Societal | **HCP**: “I think that if the child doesn’t show signs of abuse, but if a lot of obstacles are present and nobody takes the responsibility to do some- thing with these obstacles, then I think you are taking part in neglecting the child”. (30) |
| - 1. **The narrative: Listen and make it happen** | |
|  | **HCP:** ‘‘I have never had a situation where a pupil has come and given a direct account of what they have been subjected to.’’ (28)  **A^HCP^:** ‘‘The informants interpreted their professional responsi- bilities in managing suspected child maltreatment with two different roles: the supporter; and the reporter. ’ (31) |
| - 1. **Effects of disclosure** | |
| - - 1. **Positive aspects** | None |
| - - 1. **Negative aspects** | |
| - - - 1. For the survivors | **HCP:** “The problem remains the same we don’t have any law on child protection....Police will come and after investigation they will hand over the custody of child to parents.” (29) |
| - - - 1. For the healthcare professionals | **A^HCP^**: “The informants in the study pointed out deficiencies in the organization regarding support and men- toring in the disclosure process. They also raised the issue of being lonely in the reporting process and the lack of feedback from CPS. Not receiving response to their reporting increased their uncertainty in handling this type of situations.” (28)  **HCP**: ‘‘. . . we just can’t save everyone. You can’t get involved in every case.’’ (28) |
| 1. **Disclosure experienced by the survivors** | |
| - 1. **Obstacles and facilitators** | |
| - - 1. **Obstacles** | |
| - - - 1. Obstacles common to children and adults | **MS:** “I would always feel so dirty, helpless, scared, and angry. I was angry at him but mostly myself. I felt it was my fault” [age 13]. (32)  **AS:** ‘’... you tell your mum anything and I’ll belt the hell out of you, or I’ll kill you.’ Jane’s behaviour as an adult was still being governed by fear of her father. She was continually moving because ‘the thought of him coming after me is still in my mind’ » (37)  **MS:** “I waited practically years to say anything to anybody . . . because I didn’t know what to say or how to say it” [age 14]. (32)  **A^AS^**: “The first level of barriers labelled Barriers from Within, includes the following subthemes: internalization of victim- blaming; protecting oneself; and immature development at time of abuse.’’ (35) |
| - - - 1. Obstacles identified by survivors as adults, years after disclosure | **AS:** “Rod described the shame associated with his growing realisation that his experiences had been homosexual: ‘As I grew to my mid-teens and understood what had happened at that time of him taking my manhood away from me, um, that’s when I began to get the sense of embarrassment, of shame, guilt, and kept it to myself.’.’’ (37) |
| - - 1. **Facilitators of disclosure** | |
| - - - 1. Facilitators common to children and adults | **MS:** “I often do think to myself God if we actually didn’t have that argument would I have told?” (16-year-old girl). (41)  **MS:** “I just felt bad really like holding it in . . . it was just like I had to tell or it was just gonna be there for ever and ever and it’s just gonna annoy me I just had to tell. (13-year-old boy)” (41)  **A^MS^**: “ Thus, the pressure cooker effect represented a dynamic in the process of disclosure of pressure building up toward the actual telling but also characterized the nature of the telling experience.” (41) |
| - - - 1. Facilitators identified by youth, just after the disclosure | **MS:** “When something bad happens to you, you just need to talk to [someone] about it, get it out. If you got somebody who is willing to listen, and even give you their input every now and then, it makes you feel much better. And somebody to just let you know it is not your fault, you did nothing wrong.” (33) |
| - 1. **The narrative of the disclosure** | |
| 2.2.1.Just after the sexual abuse | **MS:** “My mom was really shocked and scared. She was really mad and she went told him to get out the house” (age 11) (36)  **MS:** “She started to be angry ... started to cry ... and told me why didn’t I told her about what happened” (age 6).) (36)  **MS:** “Like I never actually planned to turn round and tell somebody. I never said ‘right today’s the day I’m gonna tell somebody’” (16-year-old girl). (41) |
| 2.2.2. Long after the sexual abuse | **AS :** ‘‘Umm, so after the breakdown, I found I was having all these memories. I was being flooded, so eventually I was put in long-term therapy. I was told that’s what I needed....Otherwise, my whole life I went through blocking out, umm, reality as much as possible.’’ (38)  **A^AS^** : ‘‘Thus, their recalls illustrate a process evolving over time and along life-course inside encounters with important others, towards whom they paid attention, attunement, and adjustment whether to delay, re-try, turn towards others, withhold, or actually disclose.’’ (39) |
| - 1. **The effects of disclosure** | |
| - - 1. Positive aspects |  |
| - - - 1. Just after the disclosure | **MS:** “I think counseling is very good for me. It really helps me. I have learned from myself and my counselors a lot, I really love this place. This place is always going to be stuck in my head” (age 11) (36)  **MS:** “I’ve learned how to open up and know there are people in this world who care for me and want to listen and help me. . .. I’ve also learned to trust people and not block everyone out” [age 13] (36) |
| - - - 1. Long after the disclosure | **AS:** “This is the first time I came out with this.” (33)  **AS:** James talked about his experience disclosing to his family and how beneficial being believed and listened to was “the biggest help for me, um, was, was having them believe me and uh, being okay with talking about it.”(42) |
| - - - 1. Common to children and adults | **AS:** “I was quite nervous and embarrassed so I uh, ya know, didn’t have a lot to say but ... the counselor was definitely uh, was helpful, and just kind ... rewording things so that I could ... really understand that it really, was really not my fault » (42). |
| - - 1. Negative aspects | |
| - - - 1. Just after the disclosure | **MS:** “ I mean, the whole idea just pisses me off that here I was I couldn’t have been like, seven or eight, why would I lie about something like that? I don’t see stuff like that on TV, when I was watching Scooby Doo then. Scooby Doo didn’t teach me that. You know, I wasn’t going to lie about it and I didn’t. They didn’t believe me, and it was just pathetic.” (34) |
| - - - 1. Long after the disclosure | **A^AS^ :** « They had all experi- enced some form of disclosure-related trauma, either because nothing changed for the better or because they were not believed or supported. » (37) |
| - - - 1. Common to children and adults | **AS:** ‘... the reaction of the young person’s family to his disclosure of the abuse preoccupies him as much, if not more, than the threats that are designed to keep him quiet; will they believe me or will they believe the denials of the other? Will they really do something to help me or will they punish me for having talked about it?’ (37)  **A^AS^** : ‘Disclosure has been described as a traumatic process for the child. ’ (37) |

**AS=** Adult survivors**; MS=** Minor survivors**; HCP=** Healthcare professionals; **A^AS^=** Authors (studies with Adult survivors **A^MS^**= Authors (studies with Minor survivors); **A^HCP^=** Authors (studies with HCP)
